# Supplementary material for: HIVprotI: an integrated web based platform for prediction and design of HIV proteins inhibitors
Source: J Cheminform. 2018 Mar 9;10:12. doi: 10.1186/s13321-018-0266-y (PMC5845081; doi:10.1186/s13321-018-0266-y)
Supplement: Supplementary file 2 — Additional file 2. Source code of HIVProtI web server. [file 13321_2018_266_MOESM2_ESM.zip › HIVprotI_Source-code/hivprotI/weka-3-6-6/doc/weka/gui/graphvisualizer/GraphConstants.html]

GraphConstants


---


|  |  |  |
| --- | --- | --- |
| **Overview** \| **Package** \| **Class** \| **Tree** \| **Deprecated** \| **Index** \| **Help** \| **Weka's home** | |  |
| **PREV CLASS**   **NEXT CLASS** | **FRAMES**    **NO FRAMES**     **All Classes** |  |
| SUMMARY: NESTED \| FIELD \| CONSTR \| METHOD | DETAIL: FIELD \| CONSTR \| METHOD |  |


---


## weka.gui.graphvisualizer Interface GraphConstants

**All Known Implementing Classes:**: BIFParser, DotParser, GraphNode, GraphVisualizer, HierarchicalBCEngine

---

``` public interface GraphConstants ```

GraphConstants.java

**Version:**
:   $Revision: 1.5 $ - 24 Apr 2003 - Initial version (Ashraf M. Kibriya)

**Author:**
:   Ashraf M. Kibriya (amk14@cs.waikato.ac.nz)

---

| **Field Summary** | |
| --- | --- |
| `static int` | `DIRECTED`             Types of Edges |
| `static int` | `DOUBLE`             Types of Edges |
| `static int` | `NORMAL`             NORMAL node - node actually contained in graphs description |
| `static int` | `PLURAL_DUMMY`             PLURAL\_DUMMY node - node with more than one outgoing edge i.e. |
| `static int` | `REVERSED`             Types of Edges |
| `static int` | `SINGULAR_DUMMY`             SINGULAR\_DUMMY node - node with only one outgoing edge i.e. |

**Field Detail**

### DIRECTED

```
static final int DIRECTED
```

:   Types of Edges

    **See Also:**: Constant Field Values

---


### REVERSED

```
static final int REVERSED
```

:   Types of Edges

    **See Also:**: Constant Field Values

---


### DOUBLE

```
static final int DOUBLE
```

:   Types of Edges

    **See Also:**: Constant Field Values

---


### SINGULAR\_DUMMY

```
static final int SINGULAR_DUMMY
```

:   SINGULAR\_DUMMY node - node with only one outgoing edge
    i.e. one which represents a single edge and is inserted to close a gap

    **See Also:**: Constant Field Values

---


### PLURAL\_DUMMY

```
static final int PLURAL_DUMMY
```

:   PLURAL\_DUMMY node - node with more than one outgoing edge
    i.e. which represents an edge split and is inserted to close a gap

    **See Also:**: Constant Field Values

---


### NORMAL

```
static final int NORMAL
```

:   NORMAL node - node actually contained in graphs description

    **See Also:**: Constant Field Values


---


|  |  |  |
| --- | --- | --- |
| **Overview** \| **Package** \| **Class** \| **Tree** \| **Deprecated** \| **Index** \| **Help** \| **Weka's home** | |  |
| **PREV CLASS**   **NEXT CLASS** | **FRAMES**    **NO FRAMES**     **All Classes** |  |
| SUMMARY: NESTED \| FIELD \| CONSTR \| METHOD | DETAIL: FIELD \| CONSTR \| METHOD |  |


---
